# Targeting the BMP Pathway in Prostate Cancer Induced Bone Disease

**DOI:** 10.3389/fendo.2021.769316

**Published:** 2021-12-10

**Authors:** Desiree M. Straign, Claire L. Ihle, Meredith D. Provera, Philip Owens

**Affiliations:** ^1^ Department of Pathology, University of Colorado Anschutz Medical Campus, Aurora, CO, United States; ^2^ Cancer Biology Graduate Program, University of Colorado Anschutz Medical Campus, Aurora, CO, United States; ^3^ Department of Veterans Affairs, Research Service, Eastern Colorado Health Care System, Aurora, CO, United States

**Keywords:** bone morphogenetic protein, prostate cancer, metastasis, lytic, blastic, bone

## Abstract

From the 33,000 men in the U.S. who die from prostate cancer each year, the majority of these patients exhibit metastatic disease with bone being the most common site of metastasis. Prostate cancer bone metastases are commonly blastic, exhibiting new growth of unhealthy sclerotic bone, which can cause painful skeletal related events. Patient’s current care entails androgen deprivation therapy, anti-resorptive agents, radiation, and chemotherapy to help control the spread of the cancer but little intervention is available to treat blastic bone disease. The transforming growth factor beta (TGFβ) and bone morphogenetic protein (BMP) pathways are known to regulate bone growth and resorption of destructive lytic bone lesions, yet the role of TGFβ/BMP signaling in prostate cancer blastic vs lytic bone lesions are not fully understood. We hypothesized that to target the BMP/TGFβ pathway, a useful biomarker of bone lytic or blastic pathology would have superior response. We show distinct BMP vs. TGFβ signaling in clinical samples of human prostate cancer bone metastases with either lytic or blastic pathologies. BMPs exhibit distinct effects on bone homeostasis, so to examine the effect of BMP inhibition on healthy bone, we treated mice with the BMP receptor small molecule antagonist DMH1 and saw a modest temporary improvement in bone health, with increased trabecular bone. We next sought to use the BMP inhibitor DMH1 to treat bone metastasis engraftment seeded by a caudal artery injection of the lytic human prostate cell line PC3 in immunodeficient mice. The colonization by PC3 cells to the bone were restricted with DMH1 treatment and bone health was importantly preserved. We next proceeded to test BMP inhibition in an injury model of established bone metastasis *via* intratibial injection of the MYC-CaP mouse prostate cell line into FVBN syngeneic mice. DMH1 treated mice had a modest decrease in trabecular bone and reduced lymphocytes in circulation without affecting tumor growth. Taken together we show unique responses to BMP inhibition in metastatic prostate cancer in the bone. These studies suggest that profiling bone lesions in metastatic prostate cancer can help identify therapeutic targets that not only treat the metastatic tumor but also address the need to better treat the distinct tumor induced bone disease.

## Introduction

Tumor induced bone disease (TIBD) continues to present as a high morbidity causation in the progression of many metastatic cancers (1). Metastasis to bone is a common occurrence for cancers of the breast, lung, kidney, thyroid and especially the prostate (2). Prostate cancers have tremendously benefitted from advances in hormone, surgical and radiological interventions for treatment of the localized disease (3). Unfortunately, the consequence of this success is that patients who are living longer are now encountering relapse of castrate resistant metastatic prostate cancer (mCRPC) and older age co-morbidity factors such as diminished bone health (4). Successful treatment of patients’ cancer must frequently integrate the health of the organ site of the disease. As prostate cancer transitions from a local disease to one of the bone, care must improve the bone health. Bone mediated therapies are largely restricted to anti-resorptive medicines and rarely focus on anabolic mediated strategies (5, 6). As current molecular and cellular targets in cancer are being rapidly developed for precision oncology, a greater understanding of the pathological players in TIBD is warranted (7–9). Our group recently explored the TIBD phenotypes of mCRPC and found distinct molecular and cellular changes in bone with either osteolytic or sclerotic and osteoblastic manifestations (10). This has led to the current study to look at the osteogenic factors such as the transforming growth factor β (TGFβ) family of molecules, in particular bone morphogenetic proteins (BMPs).

BMPs are a large family of cytokine growth factors belonging to the TGFβ family with special emphasis on their osteogenesis functions (11). Originally first described by Marshall Urist in 1965 as the secreted factors capable of inducing the bone differentiation program, BMPs have widely expanded into diverse functions in development (12). Like TGFβ, BMPs have also suffered a context dependent role in cancer, the tumor microenvironment, and the surrounding tissue (13). While many tumor suppressive roles of BMPs have been described, in prostate cancer their roles have been that of tumor promoters in both the tumor and surrounding stroma (14). Unique to prostate cancers has been the elevated expression of the BMP-6 ligand as cancer progresses (15, 16). This heightened expression has also been seen with other BMP secreted ligands. As part of the unique mCRPC phenotype in bone, several studies have shown prostate cancer cell lines secretion of BMPs can stimulate osteoblast cells *in vitro* to enhance matrix deposition or promote osteogenesis programs, both of which can be reversed with BMP inhibition (17, 18).

BMPs not only act on osteoblasts to drive the health or pathology of bone, BMPs are also highly complex mediators within the bone, including regulating osteoclast maturation and function (19). BMPs have also shown distinct anatomic phenotypes based on location of the bone. BMPs share many common signaling mediators of TGFβ and Activin signaling canonically through Smad proteins with pSmad2/3 signaling controlled by TGFβ/Activin and BMPs driving activation of pSMADs 1/5/9. While TGFβ inhibitors have shown unique promise, BMP antagonists have shown mixed results, often with specific contexts unclear. We previously demonstrated that BMP small molecule inhibition could restrict mammary carcinoma metastasis to the lung by restricting BMP differentiation programs in both the tumor and microenvironment (20). The use of DMH1, a derivative of dorsomorphin, that can selectively target ALK2 and ALK3, with some activity in other kinases, can be moderately successful with lowered toxicities (21, 22). Other groups have recently shown that BMP inhibition in osteolytic disease from multiple myeloma are able to prevent TIBD (23). These and other findings set the stage for precision oncology in the metastatic bone microenvironment in the hopes that targeting BMP/TGFβ signaling in the tumor and surrounding stroma could restrict TIBD.

In this study we first investigated the canonical TGFβ/BMP signaling in lytic and blastic TIBD of mCRPC patient bone biopsies. We next sought to test the effect of a small molecule BMP receptor antagonist on the health of mouse bones as we were concerned that BMP inhibition could block normal bone formation. We then proceeded to test the requirement of BMP signaling in TIBD from a novel experimental metastasis model by caudal artery injection in mice. Finally, we then tested through intra tibial injection of mice with a syngeneic mCRPC cell line the benefit of BMP inhibition on TIBD. Our findings revealed that distinct lytic and blastic bone pathology are potential targets of the BMP pathway. With the aid of precise contextual diagnosis of mCRPC patients with TIBD, a viable treatment strategy is one that could improve bone health and restrict the progression of bone lesions.

## Materials and Methods

### Histology and Immunohistochemistry (IHC)

Deidentified IRB exempt human patient surgical specimens from bone containing prostate cancer restricted to bone without soft tissue involvement were decalcified and formalin-fixed and paraffin-embedded (FFPE) obtained from the University of Colorado Pathology Shared Resource. Ex vivo mouse tissues were harvested and immediately placed in 10% formalin and fixed for 24 hours. Formalin was replaced with 14% EDTA (Research Products International) for 5-7 days until decalcified and placed in 70% ethanol for 24 hours prior to embedding in paraffin wax. FFPE tissue blocks were sectioned at 5µm thickness with two sections per slide and mounted on plus coated microscope slides. Slides were dewaxed and rehydrated with the following sequence of washes: xylene (StatLab), 100% ethanol, 95% ethanol, 70% ethanol, 50% ethanol, and then phosphate buffered saline (PBS) (Research Products International). Heat-induced antigen retrieval was performed by microwaving slides in citrate pH6 Antigen Unmasking Solution (Vector Laboratories) in a rice cooker. Routine H&E staining was performed in Harris hematoxylin (Vector Labs). Primary antibodies for pSMAD1/5/8 (Millipore 1:100) and pSmad3 (Abcam 1:200) were used for immunohistochemistry (IHC) staining. Signal was detected by ImmPRESS polymer secondaries to appropriate host and DAB chromogen substrate (Vector Labs) and counterstained with Hematoxylin QS (Vector Labs). All bright field IHC and H&E were scanned at 40X (0.22um/pixel) magnification using a ScanScope XT System (Aperio Technologies). To quantitate IHC staining, a grid of up to five 20X images per slide was captured, avoiding excessive stroma or necrotic tissues, on an Eclipse Ni microscope (Nikon) and imported into ImageJ (U.S. National Institutes of Health) to auto-contrast images then change contrast to blue and then the threshold was set and staining was measured by percent area.

### Animals and Mouse Models of Experimental Bone Metastasis

For tumor naïve studies, twenty C57BL6/N (Taconic) males at 8 weeks of age with healthy appearance and weights over 20g were used. Mice were placed into four groups (n=5/group). Mice received a 6-week osmotic pump (Alzet 2006 #0007223), experiments in **Figures 3**, **4** used 4-week pumps (Alzet 2004 #0000298) of DMSO or DMH1 (30mg/mL) and two groups received zoledronic acid (ZA) (Sellekchem #S1314) treatment *via* IP injection (25ug/100µl). Animals were anesthetized using isoflurane 2-5%, site was prepped for a small incision at the base of the shoulders for subcutaneous implantation of the pump. Skin was closed using wound clips (BD 427631). 6 and 12 weeks from initial treatment the animals were analyzed by Dual-energy X-ray absorptiometry (DXA) while anesthetized. At week six, with pump expired, the ZA groups received an additional injection of zoledronic acid. To model bone metastasis in mice, a Caudal Artery Injection (CAI) of PC3 cells in 100ul sterile PBS were injected into the tail *via* the caudal artery as previously reported (24). NSG (Jackson Laboratories Stock#005557) male mice of at least 8 weeks and 25g weight were warmed and placed into a tube restrainer for CAI procedure where they were placed in a supine orientation to visualize the artery for efficient injections. As another model of bone experimental metastasis in mice, intra-tibial injection of tumor cells, 1x10^5^ MyC-CaP cells in 10µL PBS were injected into the tibia of FVBn (Jax Stock # 001800) male mice no younger than 8 weeks of age. Mice were bred and maintained at Rocky Mountain Regional Veterans Affairs Medical Center (protocol number CD1611M) as well as at the University of Colorado Anschutz Medical Campus (protocol number 00553). All the immune competent mice (FVBn and C57bl6) were housed together and immunodeficient animals (NSG) were house solitarily because of aggression in accordance with ARRIVE guidelines (25). All animal procedures were performed in accordance with the National Institutes of Health’s Guide for the Care and Use of Laboratory Animals and were approved by the Institutional Animal Care and Use Committees.

### DXA X-Ray, Micro-CT and IVIS

Dual-energy X-ray absorption (DXA) (Faxitron Ultrafocus DXA, Hologic, USA) was performed weekly or biweekly while animals were under anesthesia of inhaled isoflurane of 2-5%. Analysis of the femurs to quantify bone mineral content (BMC) and density (BMD) were performed by manually drawn region of interest surrounding the femur or tibia bilaterally under the bone only option. Bones were scanned with a µCT scanner (Skyscan 1276, Bruker, USA) at 70kV, 114mA with a 0.5mm aluminum filter using a pixel size of 6µm. Images were acquired at 0.6þ through 180þ, reconstructions and analysis were performed using NRecon, DataViewer and CTAn software (Bruker). ROI for trabecular bone (distal femur and proximal tibia), 2mm were analyzed 0.1 mm distal to the growth. For cortical tibial midshaft ROI; 2mm proximally from the tibia/fibula junction, 1mm was analyzed. IVIS Spectrum (Perkin Elmer) was used to image the animals with RediJect 2-DeoxyGlucosone (DG) 750 (Perkin Elmer 760561). IVIS imaging of 750nm 2-DG was performed as directed by using the wizard setting with Ex Filter 745 and Em Filter 800 (26).

### Cell Culture

The human prostate cancer cell line PC3 cell line was obtained from the American Type Culture Collection (ATCC) and cultured in DMEM (Cat#10-013-CV) Medium (Corning) 1X Antibiotic-Antimycotic (Thermofisher Scientific Cat#15240062) and 10% Fetal Bovine Serum (FBS) (Seradigm). The FVBn MYC-CaP cell line was obtained from ATCC/Dr. Austin Kirschner at Vanderbilt University and cultured in DMEM (Corning), Antibiotic-Antimycotic and 10% FBS (Seradigm). All cell lines were routinely tested for mycoplasma infection by PCR and authenticated by morphology and published growth rates available from ATCC. Additional DNA fingerprint authentication services were performed the CU Cancer Center Tissue Culture Core Facility for the PC3 cell line.

### Complete Blood Cell Quantification

Submandibular blood collection was performed prior to sacrifice, 200µl of blood was drawn using lancets (Goldenrod) (27). Blood was placed directly into a 1.3mL EDTA collection tube (Sarstedt 41.1395.105). Samples were analyzed for a complete CBC with a HemaTrue (Heska) analyzer using manufactures recommend settings.

### Statistics

Statistical analyses were performed using GraphPad Prism (version 9.2.0 for Windows; GraphPad Software Inc.) and Excel (version 2020/office 365 for Windows; Microsoft Corp.). All statistical tests used a cutoff p-value of 0.05 for significance with the Mann-Whitney test (non-parametric, one-tail and unpaired). Multiple group comparisons were analyzed by one way ANOVA in GraphPad with a Kruskal-Wallis post-test. The choice for these statistical tests were to compare significant differences in numerical values originating from similar sample types such as bone radiologic data.

## Results

### Human Metastatic Prostate Cancer in Bone Express Distinct TGFβ or BMP Signaling

**Figure 1:**
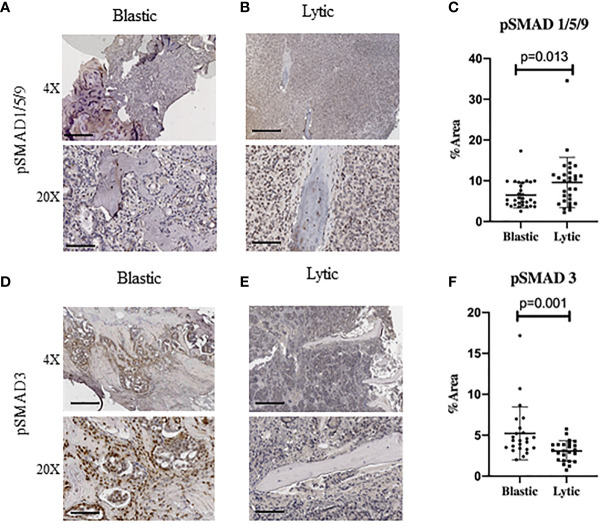
Human Metastatic Prostate Cancer in Bone Express Distinct TGFβ and BMP Signaling. Canonical BMP signaling can be detected by the nuclear translocation of BMP specific SMAD1/5/9 whereas canonical TGFβ signaling can be detected by nuclear translocation of SMAD3, which are both active when phosphorylated. **(A)** Blastic bone pathology from human patients with metastatic prostate cancer at low power (above) and high power (below) stained for pSMAD1/5/9 by IHC. **(B)** Lytic bone pathology from human patients with metastatic prostate cancer at low power (above) and high power (below) stained for pSMAD1/5/9 by IHC. **(C)** Quantification of DAB positive IHC staining by total percent area is graphed for at least five distinct fields of view for no less than 6 patients per group and analyzed for significance by Mann-Whitney U test and error bars indicate SD. **(D–F)** Blastic and lytic samples IHC stained for pSMAD3 and compared for expression between blastic and lytic pathology. Scale bars for 4X and 20X are 200µm and 50µm respectively.

To investigate whether canonical readouts of BMP and TGFβ signaling is altered in TIBD we performed Immunohistochemistry (IHC) on clinical samples containing bone with metastatic prostate cancer. These samples were previously utilized to identify cellular and molecular differences in the tumor microenvironment of TIBD based on their bone pathology, either being enriched for lytic destructive bone lesions or sclerotic and blastic bone features (10). Staining for canonical BMP signaling by hosphor-SMAD1/5/9 (pSMAD1/5/9) proteins was surprisingly low in blastic samples (**Figure 1A**). Osteolytic lesions had very strong nuclear staining in not only the tumor cells engulfing the bone but within the osteocytes and surrounding bone cells (**Figure 1B**). Staining quantitation for the six patients from both bone pathologies revealed that BMP signaling was significantly increased in lytic type disease (p=0.0125) (**Figure 1C**). We next turned to the canonical readout of TGFβ signaling hosphor-SMAD3 (pSMAD3) and were surprised to see enriched IHC staining in blastic features of metastatic prostate cancer in bone (**Figure 1D**). We noted the staining was strongly enhanced within the bone matrix and nucleus of osteoblast and osteocyte-like cells. Osteolytic lesions contained reduced nuclear pSMAD3 staining, which was largely restricted to the tumor cells and less so in the lytic bone (**Figure 1E**). Quantification of six patient samples for blastic and lytic samples revealed pSMAD3 staining was significantly increased in blastic bone lesions (p=0.0011) (**Figure 1F**).

### BMP Receptor Antagonist DMH1 Does Not Impair Healthy Bone

**Figure 2:**
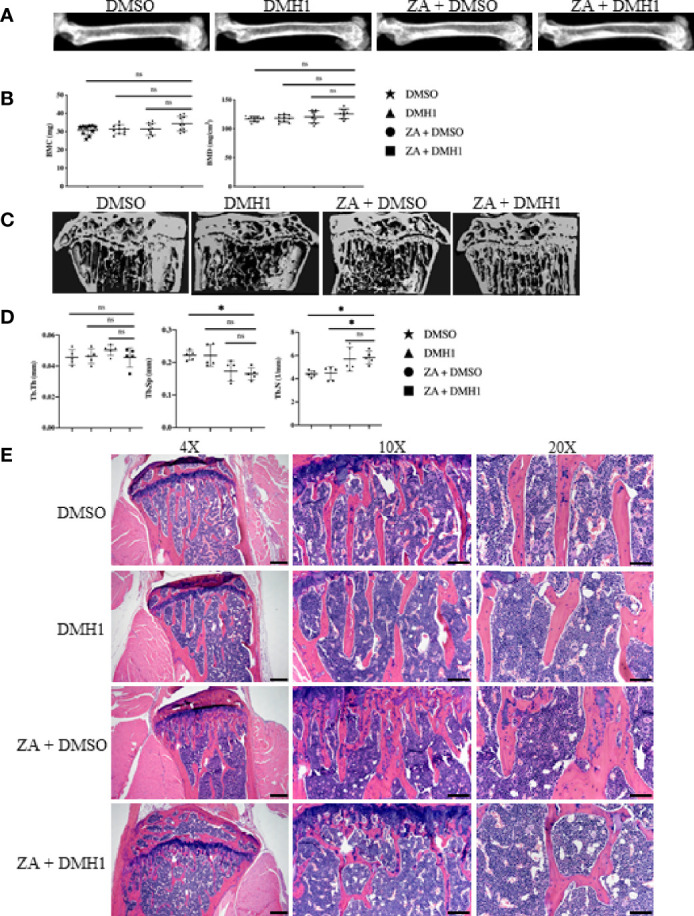
BMP Receptor Antagonist DMH1 Does Not Impair Bone. Twenty male 8 week old C57Bl6/j mice were placed into four groups (n=5 each) where they received a 6-week osmotic pump of DMSO or DMH1 alone or in addition to Zoledronic acid. 6 and 12 weeks after initial treatment with the osmotic pumps, the animals were analyzed by DXA Faxitron X-ray. At week six, with osmotic pump with DMH1 or DMSO expired, and the animals received an additional injection of zoledronic acid then monitored for another 6 weeks. **(A)** DXA representative µCT images of femurs at week 6. **(B)** BMC and BMD graphs of DXA data from week 6. **(C)** Representative image of each cohort showing the reconstruction of a cross section of the femur at week 12. **(D)** Analysis of µCT values of the femoral trabecular bone at week 12. **(E)** Representative H&E images of femur bone with emphasis in trabecular area. Scale bars for 4X, 10X and 20X are 200µm, 100µm and 50µm respectively. Statistical significance was determined using One-way ANOVA test and error bars indicate SD.

To further examine BMP signaling as a potential targeted therapy for TIBD we first wanted to test the impact on bone health in non-tumor bearing mice. Healthy C57Bl6/j male mice at 8 weeks of age were assigned into one of four groups where they all received a 6-week duration osmotic pump containing either BMP antagonist DMH1 (30ug/ml) or a control DMSO pump alone or in addition to the anti-resorptive bisphosphonate Zoledronic acid (ZA) *via* intraperitoneal (IP) injection. Mice underwent DXA X-ray at six weeks and representative images of femurs revealed no deficiencies in bone (**Figure 2A**). Using Faxitron software to restrict bone mineral content (BMC) and bone mineral density (BMD) analysis of both femurs bilaterally for the five mice in all four groups we found BMP inhibition improved BMC (p=0.04) and trended towards increased BMD (p=0.11) when combined with Zoledronic acid treatment (**Figure 2B**). In order to determine whether changes to bone health were permanent or temporary after DMH1 treatment ended, we collected the femur and tibia bones for µCT and histological analysis after the mice persisted for another six weeks past the lifetime of the osmotic pumps. Representative images from micro computed tomography (µCT) scans of the femurs indicate the changes in trabecular architecture were not significantly altered and remained healthy (**Figure 2C**). Quantification of trabecular features such as trabecular thickness (Tb.Th), trabecular spacing (Tb.Sp) and trabecular number (Tb.N) were not significantly affected by the addition of DMH1 to bisphosphonate treated mice (**Figure 2D**). Representative H&E histology of tibias highlighted the healthy architecture of mice receiving either Zoledronic acid and/or DMH1 (**Figure 2E**). Cortical image slices of mice tibias also demonstrated healthy bone (**Figure S1A**) and no appreciative changes in cortical BMD in the tibia midshaft were observed with DMH1 addition to bisphosphonate treatment (**Figure S1B**). Because BMP signaling can have a variety of effects on circulating blood we wanted to see if lasting changes to the cell populations in the peripheral blood were altered. Complete blood counts (CBCs) on mice at time of euthanasia at 12 weeks revealed normal levels of white blood cells, lymphocytes, monocytes granulocytes, and red blood cells (**Figure S1C**). However, a significant yet small increase in platelets were observed in mice treated with DMH1.

### Caudal Artery Injected Human PC3 Osteolytic Prostate Cancer Cells Have Reduced Engraftment with BMP Inhibition

**Figure 3:**
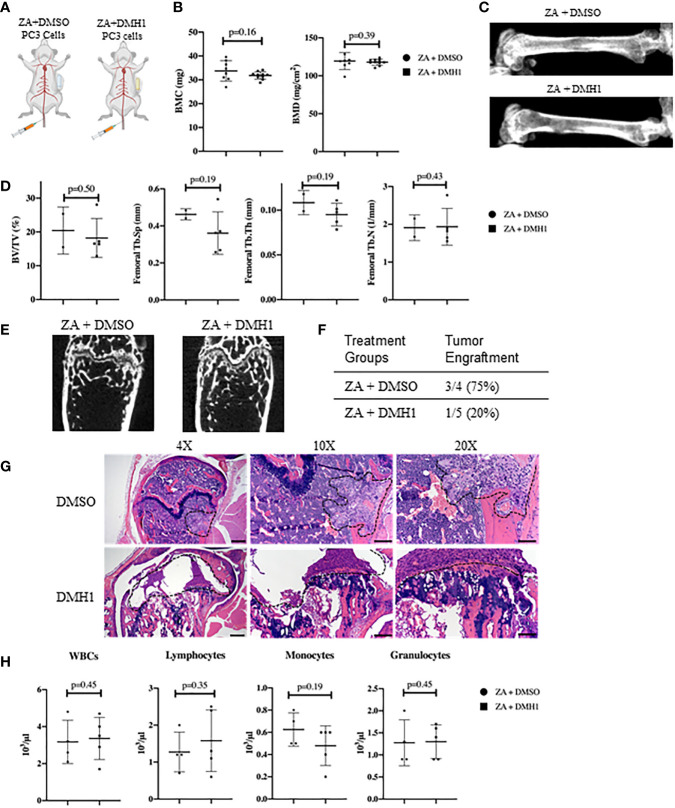
Caudal Artery Injected Human PC3 Osteolytic Prostate Cancer Cells Have Reduced Engraftment with BMP Inhibition. **(A)** Nine male 8 week old NSG mice received 50,000 PC3 cells *via* caudal artery injection. All mice received an IP injection of zoledronic acid, and subcutaneous 4 week osmotic pump implant containing DMSO (n=4) or DMH1 (n=5). **(B)** BMC and BMD measurements from femur of DXA imaging. **(C)** Representative DXA images of the femur from each treatment group. **(D)** µCT data analyzed from femur trabecular bone (DMSO n=2, DMH1 n=5 analyzed for quantification). **(E)** Representative Femoral µCT images. **(F)** Analysis of tumor incidence, with the DMSO treated group (n=4) having three mice with metastatic tumors in bone, while the DMH1 treatment (n=5) group having one mouse with metastatic tumor in bone. **(G)** H&E representative images of tumor invasion located in the femurs. **(H)** Complete Blood Count (CBC) from peripheral blood at time of euthanasia. Scale bars for 4X, 10X and 20X are 200µm, 100µm and 50mm respectively. Statistical significance was determined using a Mann-Whitney U test and error bars indicate SD.

Modeling experimental metastasis to bone is required with mCRPC as there are limited primary dissemination models from primary mouse prostate cancer (28). Approaches to colonize the bone in mouse models are typically divided into direct injection of a long bone such as a femur or tibia or injections into the heart or femoral artery can avoid the destruction and injury to trabecular bone. However, models of bone metastasis through the circulatory system face challenges for cells to engraft in the bone due to the high pressure of arterial circulation (29). A novel approach to model bone metastasis using a caudal artery injection in the tail of mice is able to utilize this arterial vasculature pressure to specifically flood the hind limbs of mice accurately and without causing an artificial injury to the bone (24). We chose to utilize the PC3 neuroendocrine like osteolytic human metastatic prostate cancer cell line with the caudal artery injection approach as the most reliable method to model vasculature mediated metastasis to the hind limbs of mice. We aimed to analyze the strong PC3 lytic phenotype in mouse TIBD and lack of tibial injury approach for bone metastasis to test the effect of BMP inhibition on bone health and metastatic engraftment. Because our data from **Figure 2** suggested DMH1 would work best in concert with ZA and many patients receive anti-resorptive therapies, all mice received an IP injection of ZA. Nine successfully injected male NSG mice received 50,000 PC3 cells in circulation *via* the tail caudal artery. The next day mice were given an IP injection of Zoledronic acid and subcutaneous implant of a 4 week duration osmotic pump carrying DMSO (n=4) or DMH1 30ug/ml (n=5) then were euthanized upon pump expiration after 4 weeks (**Figure 3A**). Mice were scanned by DXA the day prior to euthanasia and revealed no significant changes to bilateral BMC or BMD by Faxitron analysis (**Figure 3B**). Representative images were able to identify small lesions areas present in some animals (**Figure 3C**). Analysis ex-vivo by µCT for femoral bone disease revealed no significant changes to bone volume, Tb.Th, Tb.Sp or Tb.N with DMH1 treatment (**Figure 3D**). Representative femoral images from µCT revealed some absence of trabecular features equally in both treatment groups (**Figure 3E**). Validation of tumor cell engraftment came from histological confirmation of tumors in bone. Only one of the four DMSO control mice did not show evidence of disease where only one of the five DMH1 treated animals showed evidence of disease revealing that the DMH1 had restricted engraftment to femurs and tibias inspected in all animals grossly and histologically (**Figure 3F**). Representative histology confirmed the lytic nature of PC3 cells that can be seen destroying trabecular bone as well as cortical bone which led to growth outside the periosteum (**Figure 3G**). Cortical areas of bone however were not common engraftment sites and did not lead to significant changes in bone volume (**Figures S2A, B**). Analysis of peripheral blood at the time of euthanasia revealed no significant changes in blood composition from DMH1 treatment (**Figure 3H** and **Figure S2C**).

### BMP Inhibition of Established Mouse Prostate Cancer Cells Does Not Reduce Tumor Burden

**Figure 4:**
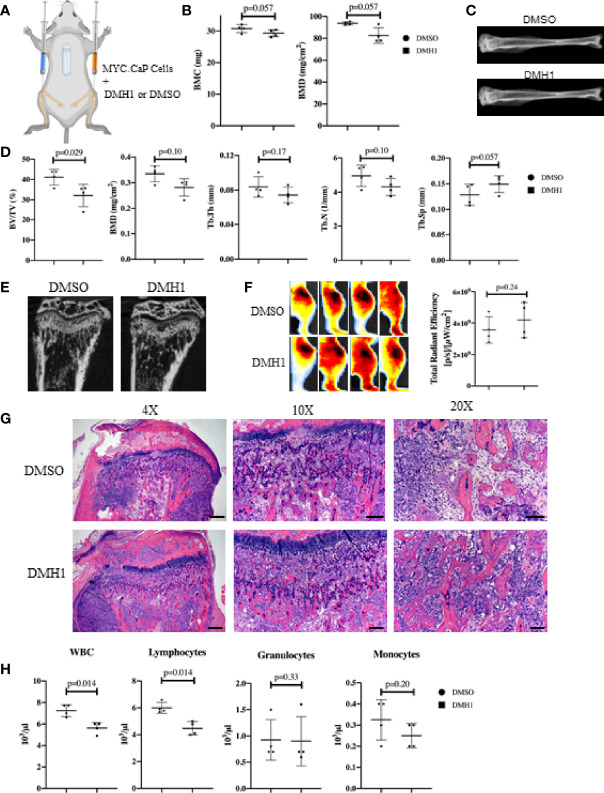
BMP Inhibition of Established Mouse Prostate Cancer Cells Does Not Reduce Tumor Burden. **(A)** Eight male 8-week-old FVBn mice received 100,000 MYC-CaP cells *via* tibial injection. All mice received a PBS injection of 10µl in the right tibia and 10µl of MYC-CaP cells injected into the left tibia. 6 days post tibial injections, mice received an IP injection of zoledronic acid and a subcutaneous 4-week osmotic pump implant containing DMSO (n=4) or DMH1 (n=4). Mice were euthanized at expiration of the pumps. **(B)** Faxitron BMC and BMD measurements derived from selecting an ROI encompassing the whole tibia. **(C)** Representative tibial images from DXA imaging. **(D)** µCT bone health quantitation data from the tibia. **(E)** Representative µCT tibial images of tumor bearing left tibia imaged *ex vivo*. **(F)** IVIS fluorescence detection using 750 2-DG mice were intravenously injected 5 hours before data acquisition at 28 days post implantation of the osmotic pump. Image shows ROIs of the tumor bearing left tibia. The graph indicates total radiant efficiency of the ROI. **(G)** Representative H&E histology slides. Scale bars for 4X, 10X and 20X are 200µm, 100µm and 50µm respectively. **(H)** Complete Blood Count (CBC) from peripheral blood at time of euthanasia. Statistical significance was determined using a Mann-Whitney U test and error bars indicate SD.

The majority of clinical bone metastases present as established lesions upon diagnosis and treatments for TIBD are still required that can target existing bone disease. This led us to test a more injury-like established model of TIBD with the tibial injection of an immune competent syngeneic cell line MYC-CaP into FVBn mice. 100,000 MYC-CaP cells were injected into the left tibia of male eight week old FVBn mice (n=8) and PBS was injected into the right tibia as a control (**Figure 4A**). Four mice received a 4 week duration osmotic pump containing DMH1 30ug/ml 6 days following tibial injection and the remaining four other mice received a DMSO osmotic pump. Mice were scanned by DXA the day prior to euthanasia and revealed significant (p=0.01) changes to BMD by Faxitron analysis of reduced density in DMH1 treated mice (**Figure 4B**). Representative images were able to identify small lesions areas present in tibias injected with MYC-CaP cells (**Figure 4C**). Analysis by µCT of tibia features demonstrated a significant loss of BMD (p=0.03), BV/TV (p=0.02) and moderate reduction in trabecular features of metastatic tibias from the DMH1 treated mice (**Figure 4D**). Analysis of cortical bone did not significantly alter the appearance or BMD in the cortical tibias (**Figures S3A, B**). Interestingly, representative images of tibias revealed significant generation of tibial and cortical bone five weeks post tumor cell injections despite the injury imposed by the intra tibial injection model (**Figure 4E**). To measure tumor burden while avoiding immune mediated rejection to Luciferase or GFP labeled MYC-CaP cells we imaged glucose uptake by IVIS with a 750nm 2-Deoxyglucosone probe and found that tumor engraftment was not significantly changed and possibly moderately increased in the DMH1 treated animals (**Figure 4F**). Histological analyses of tibial lesions revealed the metastatic progression was limited to the trabecular region injected, with no lesions progressing deep into cortical bone (**Figure 4G**) and lungs examined grossly and histologically did not indicate metastasis from the bone (data not shown). In the immunocompetent mouse model of TIBD we observed significant changes to peripheral blood composition at time of euthanasia with DMH1 resulting in significant decreases in white blood cells (p=0.002) and lymphocytes (p=0.001) (**Figure 4H**). Furthermore, peripheral blood was altered in DMH1 treated animals by significant reductions in red blood cells (p=0.001) and platelets (p=0.01) at time of sacrifice (**Figure S3C**). Finally we sought to investigate the canonical BMP and TGFβ Smad signaling response on these tumors as previously performed in clinical samples in **Figure 1**. We found that DMH1 did not reduce the canonical BMP-Smad1/5/8 signal but significantly (p=0.04) increased the staining in the bone lesions (**Figures S4A–C**). Canonical TGFβ signaling *via* pSmad3 was also significantly (p=0.03) elevated in DMH1 treated tumors, potentially highlighting the efficacy of DMH1 *in vivo* (**Figures S4D–F**).

## Discussion

The complexities of BMPs and TGFβ continue to provide unsolved mysteries in the tumor microenvironment. The bone is no exception as the understanding of bone development and diseases also contain unresolved controversy that recent studies have begun to shed light upon (30). While some similarities to the functions of tumor promoting permissive instruction from TGFβ and BMP are certainly present in TIBD, unique time and spatial requirements remain undiscovered. We currently demonstrate this phenomenon by examining pharmacologic inhibition of BMP signaling in distinct models of experimental bone metastasis with unique immune repertoires. Our previous findings that BMPR1a deletion in the myeloid LysMCre mouse model restricted primary prostate growth furthers the tumor promoting capacity that BMPs provide (31). However, in this current study the pharmacologic benefit to BMP inhibition was during engraftment of human cells lacking full immune functions (**Figure 3**). Mice with significant existing TIBD and intact immune function were not responsive to DMH1 inhibition and exhibited decreased bone and immune cells (**Figure 4**). These nuances in the role of BMP signaling in TIBD and the associated immune system in our mouse models highlight the need for precise diagnostics beyond X-ray for patients with TIBD. The potential for injury associated TIBD and the pathology of lytic or blastic disease remains poorly understood regarding therapeutic intervention (32). Today, recombinant BMP-2 is commercially used to accelerate fracture healing and is specifically indicated by the FDA for spinal fusion surgeries (33). In 2011, researchers applied for recombinant BMP-2 as a new formulation but were rejected due to fears of it as a risk for cancer (34–36). These fears were eased by independent investigations which did not find a causal link between BMP-2 and cancer risk (37). However, there are no current anabolic bone growth treatments prescribed for TIBD such as recombinant BMPs.

While the caudal artery approach for delivery of cancer cells to the long bones of mice is significantly more challenging than tibial or intra cardiac models of TIBD, we still prefer this approach for several reasons. First, it is important to have a model to avoid the injury that tibial or direct femoral injections cause, in particular since a needle puncture injury is not representative of the fractures that patients can present at time of diagnosis (38–40). Secondly, the caudal artery is superior to other non-injury vascular injections (femoral artery or intra-cardiac) because of its enrichment to the femoral long bones. We still hope that a model of experimental bone metastases that progresses from a localized bone to a soft tissue secondary metastasis such as liver or lung would be more fully developed to gauge the progression of disease demonstrated in patients. Recent advances in humanized bone scaffolds have begun to enhance the tropic mechanisms of humanized ECM in metastatic prostate cancer (41). Whichever the route of cells, it is fundamental to the nuance of cell survival prior to extravasation compared to direct inoculation into a wounded matrix. Both approaches ask significant questions required for meaningful translational targets yet a window of too much disease vs not enough time remains to be fully optimized with many models. Our experiments in **Figure 3** suggest a non-immune mediated BMP requirement for establishment of bone metastases for circulating cells. In our immune competent model where we waited until disease was much more progressed (treatment 6 days following tibial injection) was not sufficient to make meaningful impact on the growth of the tumor in the bone. Some TIBD may likely be unaltered by a systemic therapy and require more integrative care such as surgery, radiation and chemotherapies. The choice to include ZA with all animals was based on the prevalence in clinical patients with TIBD as well as the observation that DMH1 treatment had no negative effect on its increase in trabecular bone observed in **Figure 2**. DMH1 is attractive as a compound for its low toxicity and selectivity towards ALK2 and ALK3, yet this comes with low potency in micro molar *in vitro* levels. As a research *in vitro* compound it may still hold useful insight for BMP signaling, yet *in vivo* it may not be sufficient to inhibit BMP canonical activity as seen by IHC for pSmad1/5/8 in established MYC-CaP bone metastases (**Figures S4A–C**).

BMPR1a has recently been identified in breast cancer as responsible for switching osteolytic breast cells into a blastic state (42). These findings have yet to reveal the consequence of lytic and blastic pathology fully. The current mouse models of TIBD largely resides as a vicious lytic cycle but the vicious blastic cycle is not as well understood. It is quite reasonable to have biopsies of TIBD and pathologists can easily score the lytic and blastic nature of TIBD, this can also be assisted with skilled radiologists. Future investigations into TIBD need to have clear molecular targets at translationally relevant opportunities based on the bone pathology, such as BMP inhibition for lytic TIBD and TGFB inhibition for blastic TIBD. The lack of patient bone biopsies of TIBD have made it profoundly difficult to identify viable therapeutic strategies. With so much the standard of care involving radio therapy, which is a potent inducer of the TGFβ mediated fibrosis, this provides fuel to blastic fire if not carefully managed. Finally DMH1 as a drug is not a potent inhibitor of BMP signaling and while it more selectively inhibits BMP receptors than other compounds it is unlikely to achieve successful pharmacokinetics, suggesting that other strategies or future compounds that target BMP signaling may provide improved clinical potential for patients (19). This study highlights the need to begin a nuanced investigation of TGFβ family selective and specific therapeutic agents that have more fully utilized a matrix like biomarker of bone disease for successful treatments of TIBD patients (43). The timing of any therapy for TIBD must carefully assess the disease from early circulating cells to multi focal disease with soft tissue involvement and progression with clear measures of success beyond tumor growth. The management of mobility, bone health and quality of life are additional pragmatic goals that therapeutic targeting strategies can be key measures of success.

## Data Availability Statement

The raw data supporting the conclusions of this article will be made available by the authors, without undue reservation.

## Ethics Statement

Mice were bred and maintained at Rocky Mountain Regional Veterans Affairs Medical Center (protocol number CD1611M) as well as at the University of Colorado Anschutz Medical Campus (protocol number 00553). All animal procedures were performed in accordance with the National Institutes of Health’s Guide for the Care and Use of Laboratory Animals and were approved by the Institutional Animal Care and Use Committees.

## Author Contributions

PO conceived and designed experiments. DS, CI, MP, and PO performed experiments. DS and PO wrote and edited the manuscript. All authors contributed to the article and approved the submitted version.

## Funding

This work was supported by VA Grant 1KBX00002929 (PO) and NIH grant P30CA046934 for the Colorado Cancer Center Support grant.

## Conflict of Interest

The authors declare that the research was conducted in the absence of any commercial or financial relationships that could be construed as a potential conflict of interest.

## Publisher’s Note

All claims expressed in this article are solely those of the authors and do not necessarily represent those of their affiliated organizations, or those of the publisher, the editors and the reviewers. Any product that may be evaluated in this article, or claim that may be made by its manufacturer, is not guaranteed or endorsed by the publisher.

## Supplementary Material

Supplementary Figure 1 Changes to Cortical Bone and Peripheral Blood with BMP Inhibition and Bisphosphonate Treatment. **(A)** Representative µCT images of cortical slices of the tibia. **(B)** BMD of tibial midshaft µCT with n=5 per group. **(C)** 200 µl of peripheral blood from study mice was collected at the time of sacrifice for complete blood count analysis. White Blood Cells (WBC), Lymphocytes, Monocytes, Granulocytes, Red Blood Cells (RBC), Hemoglobin (HGB), Platelets (PLT) and Hematocrit (HCT) were measured from peripheral blood. Statistical significance was determined using an unpaired one-tailed t test (Mann-Whitney) error bars indicate SD.

Supplementary Figure 2 Changes to Cortical Bone and Peripheral Blood with Treatment of BMP Inhibition of Caudal Artery Injected PC3 Cells. **(A)** µCT image of a cortical slice of the tibial midshaft taken from the ROI. **(B)** Bone volume/tissue volume percentage histogram showing no significant difference between both treatment groups (n=3 ZA + DMSO, n=5 ZA + DMH1). 1mm of cortical bone was analyzed. **(C)** A complete blood count (CBC) was performed at the endpoint of the study upon submandibular collection of 200µl of peripheral blood to quantify Red blood cells (RBC), hemoglobin (HGB), platelets (PLT) and hematocrit (HCT). Statistical significance was determined using Mann-Whitney U test and error bars indicating SD.

Supplementary Figure 3 Changes to Cortical Bone and Peripheral Blood with Treatment of BMP Inhibition of Tibial Injected MYC-CaP Cells. **(A)** Representative image of mCT of MYC-CaP tumor bearing animals from the left midshaft tibia. **(B)** Bone mineral density (BMD) of the region of interest (ROI) of tibial cortical bone. ROI’s analyzed was offset by 2mm from the tibia/fibula junction and selected 1mm (100 slices) as the ROI. **(C)** CBC data showing changes in peripheral blood for red blood cells (RBCs), Hemoglobin (HGB), Platelets (PLT) and Hematocrit (HCT). DMSO group n=4, DMH1 n=4. Statistical significance was determined using Mann-Whitney U test and error bars indicating SD.

Supplementary Figure 4 Mouse Metastatic Prostate Cancer in Bone Express Heterogenous TGFβ and BMP Signaling. Canonical BMP signaling can be detected by the nuclear translocation of BMP specific Smad1/5/8, whereas canonical TGFβ signaling can be detected by nuclear translocation of Smad3, which are both active when phosphorylated. **(A)** DMSO treated mice with MYC-CaP tibial injected bone lesions stained for pSmad1/5/8 IHC. **(B)** DMH1 treated mice with MYC-CaP tibial injected bone lesions stained for pSmad1/5/8 IHC. **(C)** Quantification of DAB positive IHC staining by total percent area is graphed for at least five distinct fields and four mice per group and analyzed for significance by Mann-Whitney U test and error bars indicate SD. **(D–F)** Mouse tibia injected with MYC-CaP cells and treated with DMSO or DMH1 with IHC stained for pSmad3 and quantitated. Scale bars for 20X objective are 50µm black bars in representative images.
