# Supplementary figures and images for: Targeting the BMP Pathway in Prostate Cancer Induced Bone Disease
Source: Front Endocrinol (Lausanne). 2021 Dec 10;12:769316. doi: 10.3389/fendo.2021.769316 (PMC8702552; doi:10.3389/fendo.2021.769316)

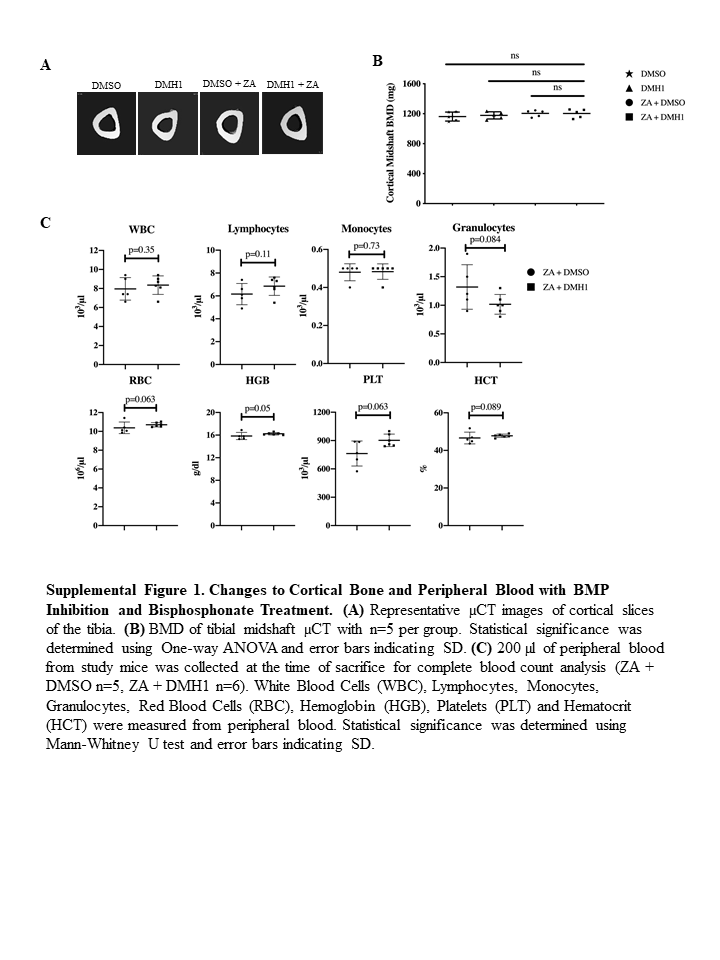

Supplement: Supplementary Figure 1 — Changes to Cortical Bone and Peripheral Blood with BMP Inhibition and Bisphosphonate Treatment. (A) Representative µCT images of cortical slices of the tibia. (B) BMD of tibial midshaft µCT with n=5 per group. (C) 200 µl of peripheral blood from study mice was collected at the time of sacrifice for complete blood count analysis. White Blood Cells (WBC), Lymphocytes, Monocytes, Granulocytes, Red Blood Cells (RBC), Hemoglobin (HGB), Platelets (PLT) and Hematocrit (HCT) were measured from peripheral blood. Statistical significance was determined using an unpaired one-tailed t test (Mann-Whitney) error bars indicate SD. [file Image_1.tif]

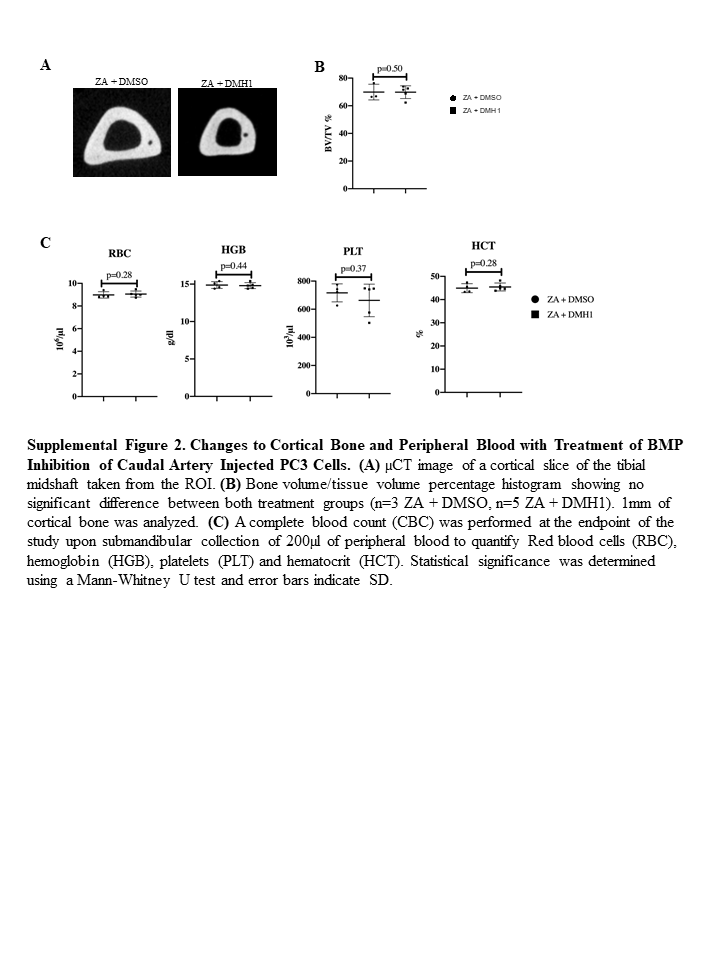

Supplement: Supplementary Figure 2 — Changes to Cortical Bone and Peripheral Blood with Treatment of BMP Inhibition of Caudal Artery Injected PC3 Cells. (A) µCT image of a cortical slice of the tibial midshaft taken from the ROI. (B) Bone volume/tissue volume percentage histogram showing no significant difference between both treatment groups (n=3 ZA + DMSO, n=5 ZA + DMH1). 1mm of cortical bone was analyzed. (C) A complete blood count (CBC) was performed at the endpoint of the study upon submandibular collection of 200µl of peripheral blood to quantify Red blood cells (RBC), hemoglobin (HGB), platelets (PLT) and hematocrit (HCT). Statistical significance was determined using Mann-Whitney U test and error bars indicating SD. [file Image_2.tif]

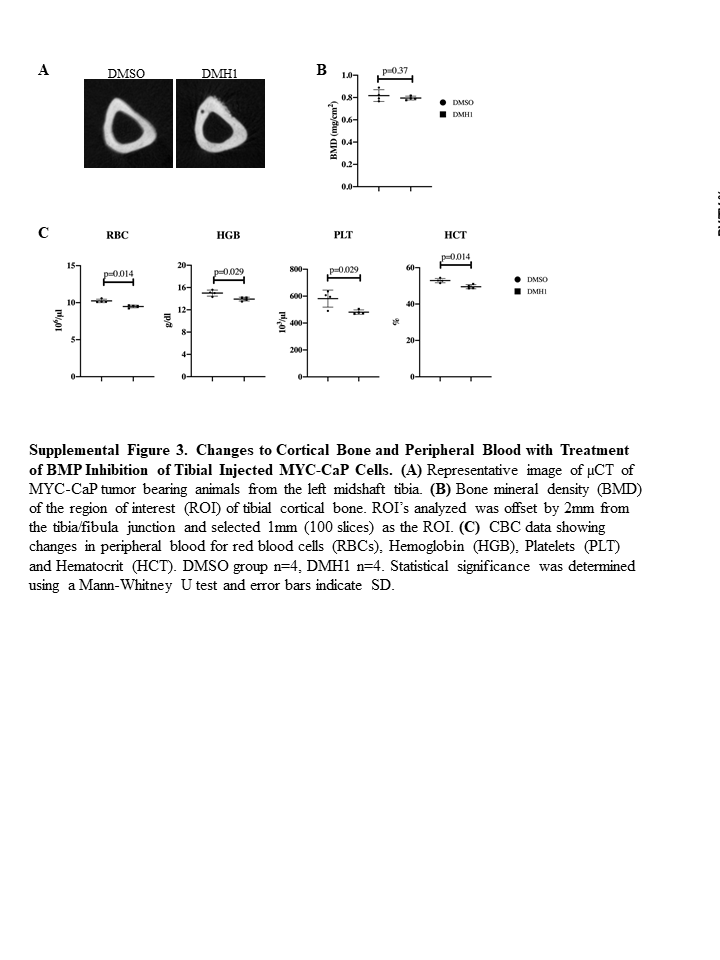

Supplement: Supplementary Figure 3 — Changes to Cortical Bone and Peripheral Blood with Treatment of BMP Inhibition of Tibial Injected MYC-CaP Cells. (A) Representative image of mCT of MYC-CaP tumor bearing animals from the left midshaft tibia. (B) Bone mineral density (BMD) of the region of interest (ROI) of tibial cortical bone. ROI’s analyzed was offset by 2mm from the tibia/fibula junction and selected 1mm (100 slices) as the ROI. (C) CBC data showing changes in peripheral blood for red blood cells (RBCs), Hemoglobin (HGB), Platelets (PLT) and Hematocrit (HCT). DMSO group n=4, DMH1 n=4. Statistical significance was determined using Mann-Whitney U test and error bars indicating SD. [file Image_3.tif]

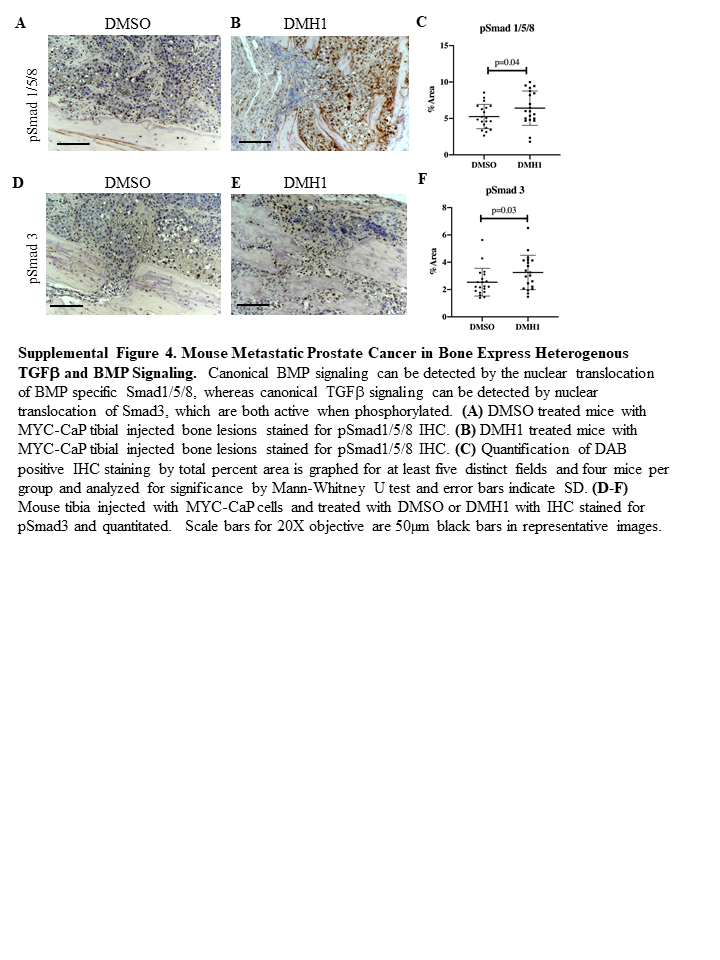

Supplement: Supplementary Figure 4 — Mouse Metastatic Prostate Cancer in Bone Express Heterogenous TGFβ and BMP Signaling. Canonical BMP signaling can be detected by the nuclear translocation of BMP specific Smad1/5/8, whereas canonical TGFβ signaling can be detected by nuclear translocation of Smad3, which are both active when phosphorylated. (A) DMSO treated mice with MYC-CaP tibial injected bone lesions stained for pSmad1/5/8 IHC. (B) DMH1 treated mice with MYC-CaP tibial injected bone lesions stained for pSmad1/5/8 IHC. (C) Quantification of DAB positive IHC staining by total percent area is graphed for at least five distinct fields and four mice per group and analyzed for significance by Mann-Whitney U test and error bars indicate SD. (D–F) Mouse tibia injected with MYC-CaP cells and treated with DMSO or DMH1 with IHC stained for pSmad3 and quantitated. Scale bars for 20X objective are 50µm black bars in representative images. [file Image_4.tif]
